# Refractory Right Coronary Artery Spasm: A Case Report

**DOI:** 10.7759/cureus.7034

**Published:** 2020-02-18

**Authors:** Siamac Yazdchi, Santosh Desai, Janelle Rodriguez, Meghanaprakash Hiriyur Prakash, Ismail A. H Bokhari

**Affiliations:** 1 Internal Medicine, Banner - University Medical Center Phoenix, Phoenix, USA; 2 Interventional Cardiology, Cardiovascular Associates of Mesa, Phoenix, USA; 3 Cardiology, Banner - University Medical Center Phoenix, Phoenix, USA; 4 Cardiology, Banner - University Medical Center Phoenix, Phoenix, USA; 5 Cardiology, Kingman Regional Medical Center, St. George, USA

**Keywords:** coronary vasospasm, coronary stenting

## Abstract

Herein, we report a case of severe coronary spasm during cardiac catheterization refractory to medical management. Although this condition is usually managed with vasodilating agents, our patient ultimately required placement of stents.

## Introduction

Coronary vasospasm is a common cause of myocardial infarction with no obstructive coronary atherosclerosis (MINOCA) syndrome [1]. Intra-procedural coronary spasm often resolves with the administration of vasodilators. A case of severe coronary spasm during cardiac catheterization refractory to medical management was encountered and ultimately managed by the placement of coronary stents. This case highlights an unusual management strategy for a patient with refractory coronary spasm. 

## Case presentation

The patient is a 52-year-old female with a past medical history of hypertension, chronic obstructive pulmonary disease, tobacco use, and polysubstance abuse. She had sustained a prior pulmonary embolism and was on anticoagulation. The patient presented with exertional angina in an outpatient setting. Her evaluation included a pharmacologic nuclear stress test which revealed anterior wall ischemia. She was referred for cardiac catheterization. 

**Figure 1:**
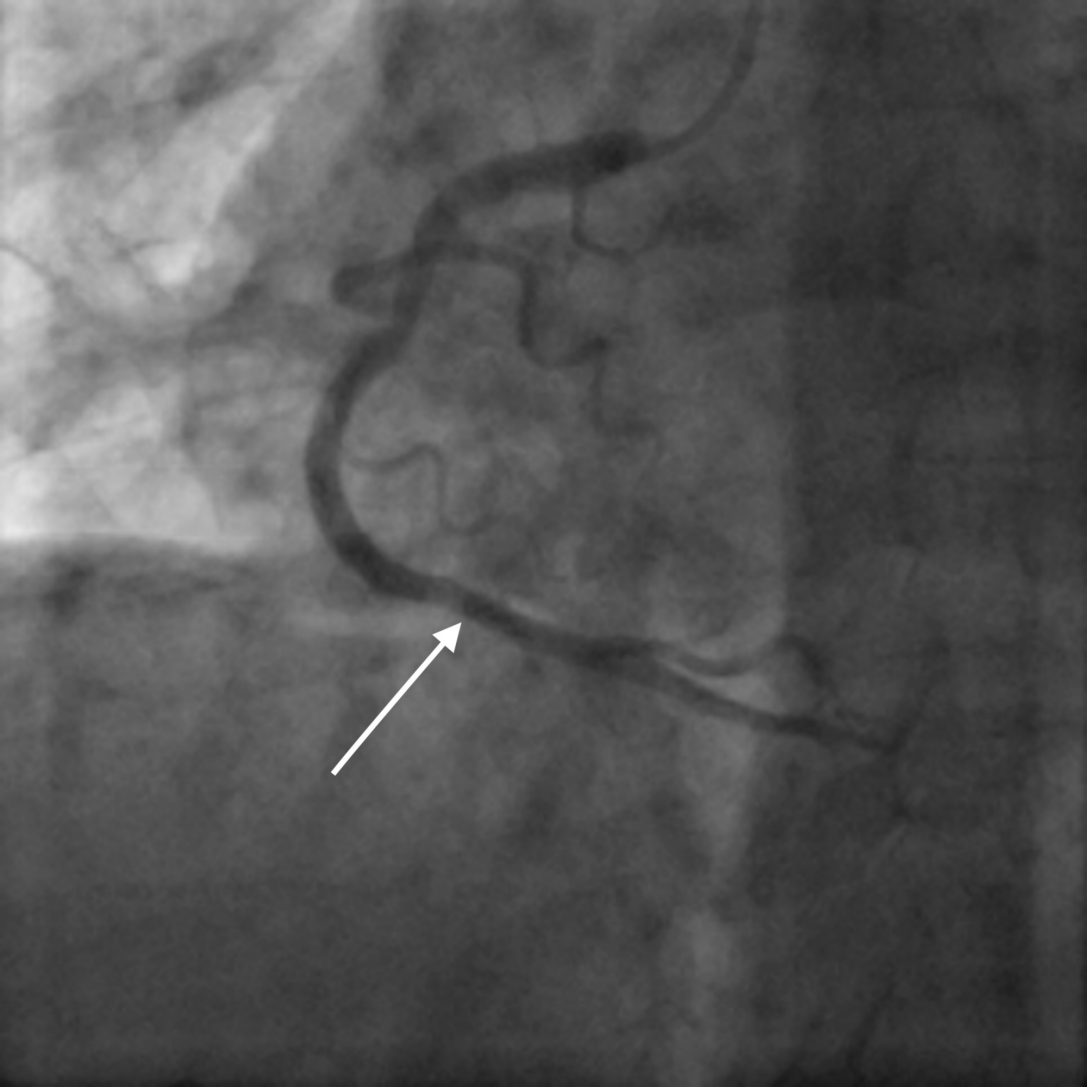
Patent right coronary artery

Right radial artery and right antecubital vein access were obtained for the left and right heart catheterization (LHC and RHC). Coronary angiography revealed coronary arteries without the disease (Figure 1, Video 1). After the right heart catheterization was completed, the patient started complaining of chest pain associated with ST-segment elevation in leads II and aVF on the monitor. The patient was uncomfortable and hypertensive. She was started on intravenous nitroglycerin which did not relieve her chest pain nor the ST-segment elevation noted in the inferior leads. An emergent echocardiogram was performed and did not show any pericardial effusion. 

**Video 1:** Right coronary artery angiography

Given the persistent symptoms and ST-segment changes in the inferior leads, a decision was made to interrogate the right coronary artery again. Right coronary angiography was performed revealing severe spams from the proximal segment to the mid-vessel with no flow distally (Video 2).

**Video 2:** Right coronary artery spasm

Intracoronary nitroglycerin and nitroprusside were administered with no significant improvement. The patient was then started on nicardipine intravenously to control her blood pressure, and the decision was made to proceed with mechanical intervention. A 2.5 mm semi-compliant balloon was advanced into the right coronary artery and multiple inflations were performed. Intravascular ultrasound was performed revealing a 4 mm reference vessel size without plaque, thrombus, or dissection. 

An intra-aortic balloon pump was inserted to augment diastolic coronary filling; however, the patient continued to have severe retrosternal chest pain accompanied by ST-segment elevation and continued evidence of spasm. 

After waiting for several minutes, a decision was made to place coronary stents to combat the refractory spasm. The risk for myocardial necrosis from the interruption of blood flow to the distal right coronary artery (RCA) and its branches seemed greater than the risk for percutaneous coronary intervention (PCI). A 3.5 mm x 32 mm stent was placed in the distal right coronary artery, a 4 mm x 38 mm stent was placed in the middle portion of the vessel, and another 4 mm x 32 mm stent was deployed to cover the proximal and distal portions of the artery (Video 3). 

**Video 3:** Right coronary artery post-percutaneous coronary intervention

The patient's symptoms resolved quickly after stenting. She was monitored closely in the Intensive Care Unit. She made an uneventful recovery and was discharged home. 

## Discussion

Coronary spasm was first demonstrated by Gensini in 1962 [2]. Changes in autonomic activity, as well as the use of illicit drugs (such as cocaine and amphetamines), have been identified as possible culprits. Guidewire or balloon manipulation at the time of percutaneous coronary intervention are among the most common causes of this condition today [3-5]. 

The mechanisms for catheter-induced spasm are not completely understood, but anatomic characteristics and myogenic reflexes caused by mechanical stimulation of the RCA may be involved [6]. The main treatment for this condition is a combination of calcium antagonists [7]. The use of beta-blockers is traditionally avoided, given their potentially detrimental effect of limiting beta-receptor-mediated dilatation and promoting unopposed alpha-adrenergic coronary vasoconstriction [8]. Donatelli et al. described a case of coronary artery spasm six hours after coronary bypass grafting, which was successfully managed with an intracoronary infusion of isosorbide dinitrate and intravenous nifedipine [9]. 

PCI is not routinely indicated for patients with focal spasm and minimal coronary disease. However, PCI may be helpful if a significant coronary obstruction is thought to be a potential trigger for focal spasm. Stent placement may be an effective therapy for patients with medically refractory vasospasm associated with mild to moderate coronary disease [10-11].

## Conclusions

The unique and interesting features of this case report making it worthy of consideration are 1) the refractoriness of the spasm to medical therapy with vasodilators, 2) the distance of the spasm from the catheter manipulation at the ostium of the RCA, and 3) the intravenous ultrasound findings of no atherosclerosis in the RCA, a finding that is different from other cases of variant angina encountered in the literature. To our knowledge, this is a unique case of coronary spasm with normal coronaries intractable to medical therapy treated with stents. 
